# Formulation optimization and synergistic effects of flocculation–solidification–vacuum preloading on sludge treatment

**DOI:** 10.1038/s41598-025-96752-7

**Published:** 2025-04-09

**Authors:** Lufan Li, Hongen Wu, Xukun Yang, Jianwen Wei, Yunliang Cui

**Affiliations:** 1 School of Engineering, Hangzhou City University, Hangzhou, Zhejiang China; 2 PowerChina HuaDong Engineering Corporation Limited, Hangzhou, Zhejiang China; 3 School of Civil Engineering and Architecture, Anhui University of Science and Technology, Huainan, Anhui China

**Keywords:** Sludge, Flocculation, Solidification, Vacuum preloading, Rice husk ash, Civil engineering, Mechanical properties

## Abstract

Rapid infrastructure development generates large volumes of high-water-content sludge, creating an urgent need for efficient recycling and management strategies. This study introduces the flocculation–solidification–vacuum preloading (FSVP) method to enhance dewatering efficiency and strength development, facilitating subsequent mechanical construction requirements. To enhance solidification and reduce cement consumption, the response surface method was used to determine the optimal composite curing agent, which consists of 53% cement, 32% rice husk ash, and 15% sodium silicate. Vacuum dewatering was applied to sludge samples treated with different flocculants and curing agents to assess their synergistic effects on soil improvement. The mixed flocculant of polyaluminum chloride and anionic polyacrylamide significantly increased the micropore content and compactness, with pore sizes primarily concentrated around 0.01 μm. While the flocculant facilitated efficient drainage, required unconfined compressive strength could only be achieved with the further addition of a curing agent. The optimal composite curing agent formulation induced hydration and pozzolanic reactions, filling larger pores with cementitious materials and enhancing soil strength. As a result, the vane shear strength reached 58 kPa and unconfined compressive strength reached 365 kPa at 7 days, further increasing to 586 kPa at 28 days.

## Introduction

With the rapid development of infrastructure construction, a large amount of waste sludge is being generated and primarily disposed of through on-site burial or stockpiling^1^. However, sludge contains heavy metal ions and other toxic substances, posing a risk of environmental contamination^2,3^. To achieve significant volume reduction and resource reutilization, vacuum preloading technology has gained widespread recognition due to its cost-effectiveness, simplicity, and efficient dewatering performance^4,5^.

Despite its advantages, vacuum preloading has notable limitations. During the dewatering process, fine soil particles in the sludge tend to accumulate near the drainage board, leading to membrane clogging and significantly reducing dewatering efficiency^6^. Additionally, vacuum pressure can only remove free water^7^, with limited effectiveness in extracting bound water^8^, resulting in inadequate soil consolidation that fails to meet engineering requirements. To mitigate these issues, flocculants are commonly applied to aggregate fine soil particles into larger floc structures. Commonly used flocculants include inorganic salt coagulants, such as ferric and aluminum salts, as well as synthetic organic polymeric flocculants, primarily polyacrylamide (PAM) and its derivatives. Studies have shown that when inorganic and organic flocculants are combined in specific proportions, they can enhance the flocculation effect and reduce flocculant dosage, such as PAM-polyaluminum chloride (PAC) and PAM-FeCl_3_^2,9^. However, the bearing capacity of the flocculated soil still remains very low^10,11^.

To better improve the mechanical properties of flocculated sludge, researchers have introduced curing agents such as cement^12,13^. This has led to the development of a novel treatment method, flocculation-solidification-vacuum preloading (FSVP) method, which integrates conventional sludge flocculation conditioning technology^11,14^, cement solidification technology^15,16^, and vacuum preloading technology^17,18^. Flocculation alters the structural arrangement of soil and cement particles, bringing them into closer contact and promoting the physical sedimentation/consolidation process of the sludge. The charge neutralization and bridging adsorption effects of flocculants promote the aggregation of soil particles into large flocs, forming a structure that is stronger and more cohesive compared to the original soil^14^. Additionally, during the solidification process, cementitious materials produced by hydration and pozzolanic reactions exhibit considerable strength, effectively binding the sludge^15,18^.

In the FSVP method, a high binder content exceeding 20% is typically required. Conventional curing agents composed solely of cement exhibit limited solidification efficiency in high-water-content sludge (> 100%), failing to achieve the required strength for embankment fill^12,17^. To address this challenge, researchers have explored the partial replacement of cement with solid waste materials to develop composite curing agents^12,19–21^, such as quicklime and fly ash^12^. In industrial applications, the formulation of curing agents must balance mechanical performance, cost efficiency, and environmental impact. This has driven researchers to seek alternative materials that are not only highly reactive but also cost-effective and abundantly available.

Rice husk ash (RHA), a byproduct of biomass power plants, is produced in large quantities yet remains largely underutilized. Due to its high content of reactive SiO_2_, RHA exhibits properties comparable to silica fume^22,23^, making it a promising candidate for subgrade stabilization^24,25^. Its pozzolanic activity can be effectively enhanced through alkali activation or the addition of calcium-based materials, promoting the formation of C-S-H gel, which fills pores and improves mechanical performance^26^. Moreover, RHA possesses high porosity, low relative density, and a large specific surface area^27^, enabling it to rapidly absorb water at early ages and gradually release it during the later curing stage^28,29^.

Although RHA theoretically exhibits pozzolanic activity and physical water absorption capacity, the strength development and optimal mix proportion of composite curing systems incorporating RHA, alkali activation, and cement have yet to be experimentally validated and optimized. Additionally, the synergistic effects of composite curing agents with flocculation and vacuum preloading under high water content conditions remain insufficiently explored.

This study optimized the formulation of a composite curing agent by partially replacing cement with RHA and incorporating sodium silicate (SS) to enhance its reactivity, utilizing the response surface method. Furthermore, the effectiveness of the FSVP treatment was assessed based on drainage performance, surface settlement, pore water pressure, vane shear strength, and unconfined compressive strength. From a microstructural perspective, pore structure analysis further validated the dewatering efficiency.

## Methodology

### Raw sludge and chemicals

**Fig. 1:**
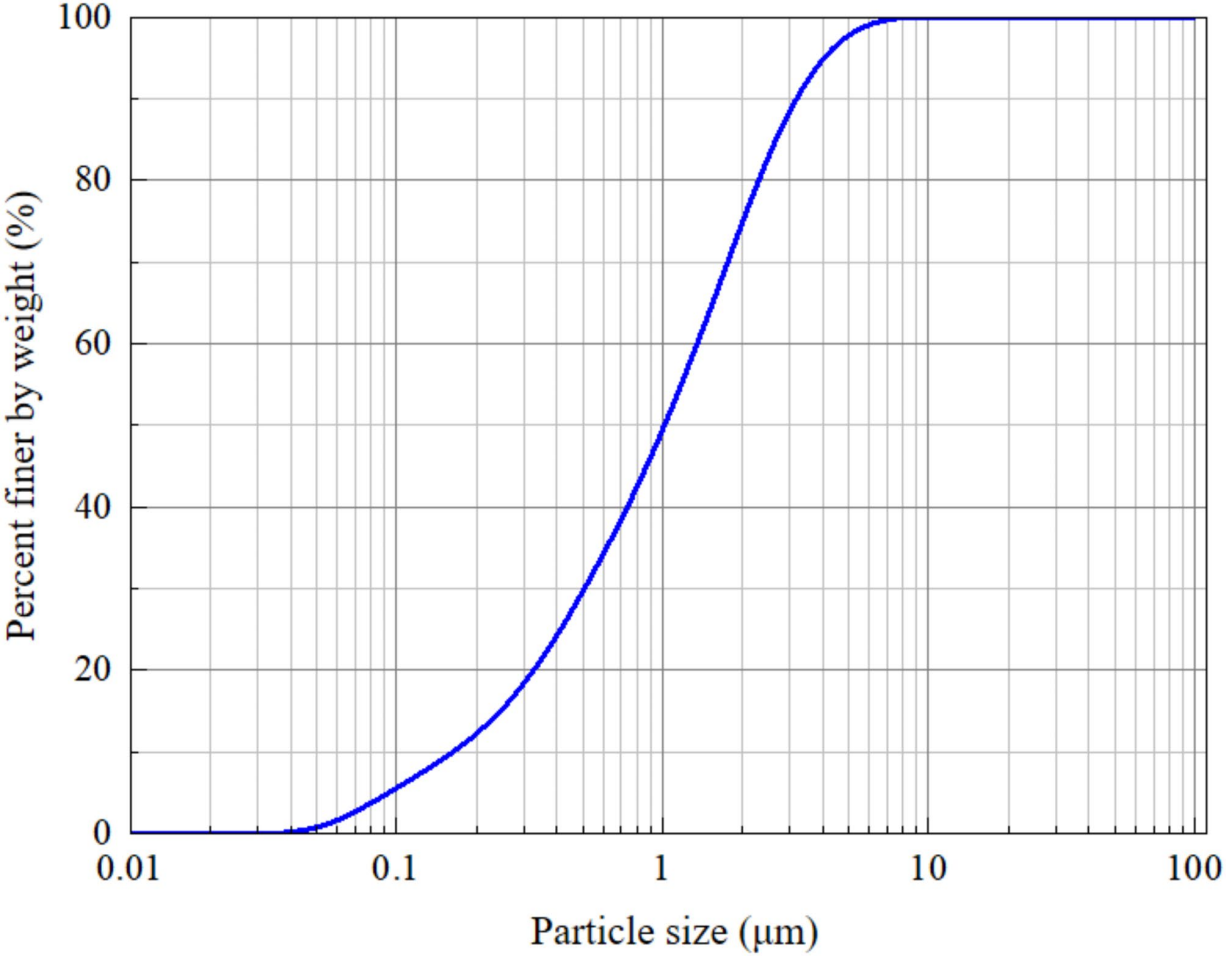
Particle size distribution of sludge.

The sludge samples, collected from a river dredging project in Hangzhou, Zhejiang, China, had a high moisture content of 93% and exhibited a flowable consistency. The particle size distribution is shown in Fig. 1, and the initial physical properties of the sludge are summarized in Table 1.

**Table 1:** Physical properties of sludge.

Moisture content (%)	Relative density	Dry density (g cm^−3^)	Liquid limit	Plastic limit	Electrical conductivity (m s cm^−1^)	pH
93.62%	2.46	0.821	53.56%	36.85%	0.103	7.2

**Fig. 2:**
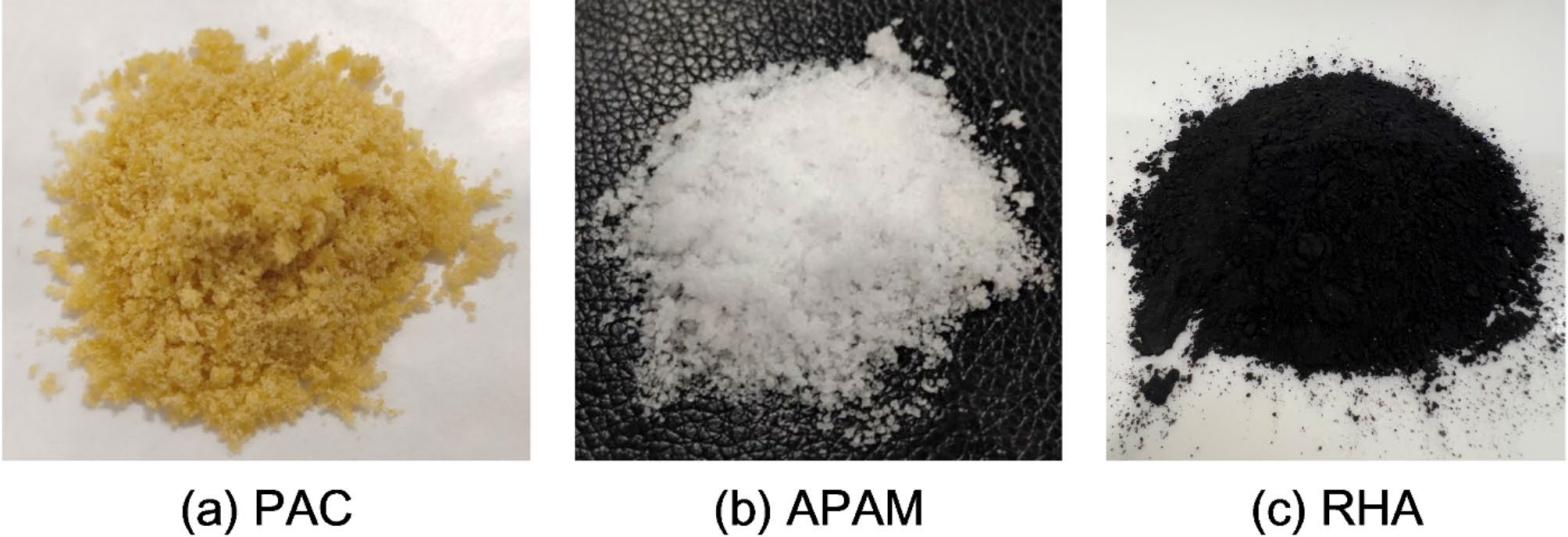
PAC, APAM, and RHA used in this study.

Polyaluminum chloride (PAC) and anionic polyacrylamide (APAM) with a molecular weight of 10 million were selected for flocculation treatment, as presented in Fig. 2a,b. P.O. 42.5 ordinary Portland cement (OPC), RHA, and SS were selected as the key components in the formulation of the composite curing agent. RHA was the solid waste product generated from the combustion of rice husks at 800 °C in power plants, which appears as a black powder (Fig. 2c) with a carbon content ranging from 5 to 15%. SS was produced by Sinopharm Chemical Reagent Co., Ltd., arrives as white powder with a sodium silicate content of no less than 99.8%. The silica modulus of sodium silicate was maintained at 1.0 to ensure consistency in the chemical composition and reactivity of the alkaline activator.

### Design of composite curing agents

#### Response surface method design

The response surface method is an efficient technique for identifying the optimal mix proportion^24^. In this design, *x*_*i*_ represents the proportion of the *i*th ingredient (where *i* = 1, 2, 3). The proportions are positive, and the sum of all ingredients equals 100%. The corresponding constraints are presented in Eq. (1). The response variable *y* is the unconfined compressive strength of the treated sludge, with three curing ages of 7 days, 14 days, and 28 days, considered as performance indicators.

1 $$\left. {\begin{array}{*{20}{c}} {{x_i} \geqslant 0~\left( {i=1,~2,~3} \right)} \\ {\mathop \sum \limits_{{i=1}}^{p} {x_i}={x_1}+{x_2}+{x_3}=1} \end{array}} \right\}$$

The contents of OPC, RHA, and SS are denoted as A, B, and C, with corresponding codes *x*_*1*_, *x*_*2*_, and *x*_*3*_. Detailed mix proportion of composite curing agents are presented in Table 2. Some code values were repeated to validate the stability and improve the accuracy of model.

**Table 2:** Mix proportion of composite curing agents.

No.	Code value			Proportion of ingredients (%)		
	OPC	RHA	SS	OPC	RHA	SS
1	0.61	0.39	0	15.33	9.67	0
2	0.51	0.17	0.32	12.65	4.35	8
3	0.73	0.24	0.03	18.17	6.08	0.74
4	0.73	0.13	0.14	18.16	3.31	3.53
5	0.51	0.17	0.32	12.65	4.35	8
6	0.65	0.03	0.32	16.22	0.78	8
7	0.79	0	0.21	19.71	0	5.29
8	0.60	0.22	0.18	14.91	5.56	4.53
9	0.79	0	0.21	19.71	0	5.29
10	1	0	0	25	0	0
11	0.73	0.13	0.14	18.16	3.31	3.53
12	0.73	0.24	0.03	18.17	6.08	0.74
13	0.87	0.13	0	21.75	3.25	0
14	0.48	0.52	0	12	13	0
15	0.49	0.35	0.16	12.31	8.67	4.02
16	0.49	0.35	0.16	12.31	8.67	4.02

#### Sample preparation

To align with the practical requirements of engineering applications, the samples were prepared in a wet state. To minimize experimental variation, the water content of the sludge was standardized at 100% prior to testing. The sludge was then sieved through a 2 mm stainless steel mesh to remove impurities and stored in sealed containers to preserve its uniformity. Based on the mix design in Table 2, the curing agent was prepared and incorporated into the sludge, followed by the addition of flocculants (1.5% PAC-APAM), and stirring for 3 min to ensure uniformity.

**Fig. 3:**
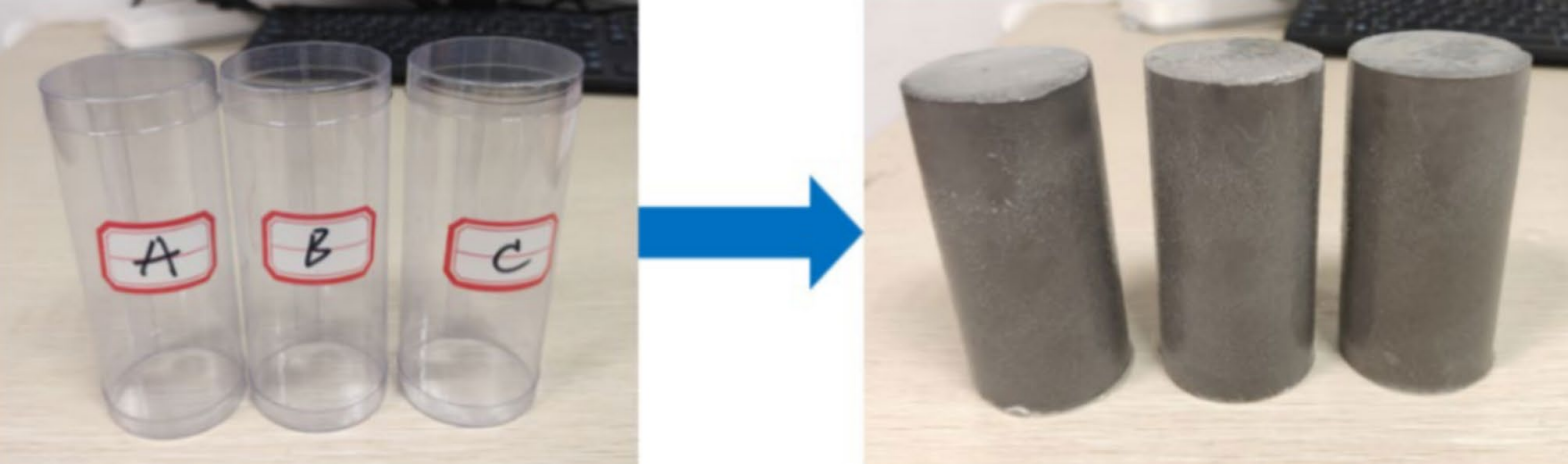
Sample preparation for composite curing agent study.

PVC cylindrical molds with an inner diameter of 50 mm and a height of 100 mm were employed, each fitted with top and bottom sealing caps to ensure airtight containment, as shown in Fig. 3. The inner walls of the mold were pre-coated with Vaseline. The mixed sludge was placed into the PVC mold in three layers, each layer being vibrated for 3 min on a vibrating table to ensure uniformity and density. Three parallel samples were prepared for each group following the same procedure. After filling, the molds were tightly sealed.

After 48 h of resting, the molds were removed. Any excess material at the top was scraped off with a geotechnical knife to achieve a smooth surface. The test block was then placed in a constant temperature and humidity curing box set at 20 ± 5 °C, where it was cured until the required testing age. Following the curing period, unconfined compression tests were conducted in accordance with the Chinese national standard *GB/T 50123-2019 ‘Standard for Geotechnical Testing Methods’*.

### Vacuum dewatering model test

#### Experimental schemes

Based on our previous research4444.

#### Vacuum dewatering experiment setup

**Fig. 4:**
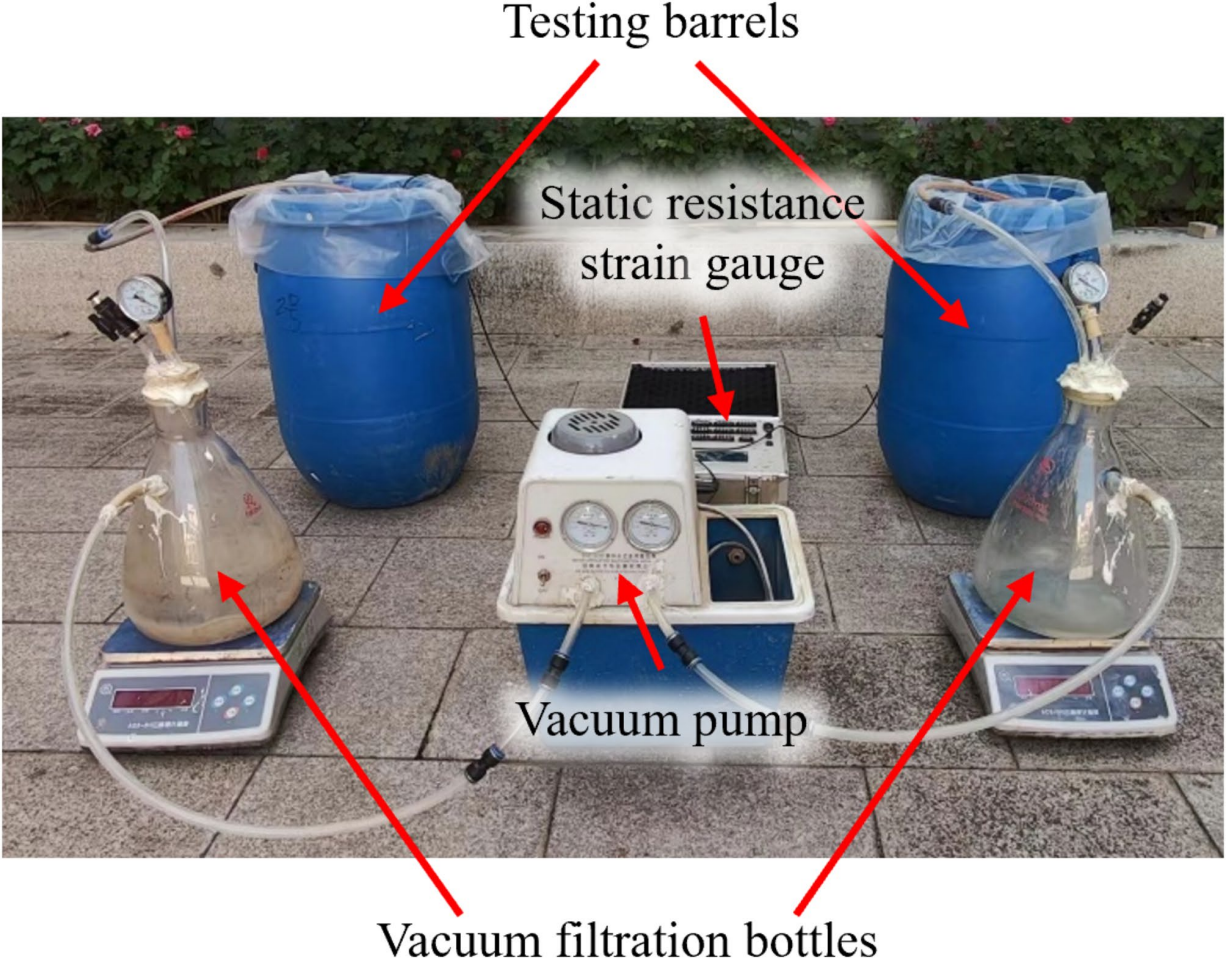
Vacuum dewatering model device setup.

The vacuum dewatering experiment setup is presented in Fig. 4. The water content of the sludge was adjusted to 140% and mixed for 5 min to ensure uniformity. The pH of M2-M7 group was first adjusted to 4.2, then flocculants and curing agents were added as per the test plan. Special attention should be given to the M3 group, where PAC was added first, followed by APAM. For M6 and M7 groups, the curing agent were added first, followed by the flocculant, and the mixture stirred for 3 min to ensure even distribution. After mixing, the sludge was carefully poured into the testing barrel with proper placement of drainage.

The pore water pressure gauge (range 0–100 kPa) was embedded 0.2 m above the bottom of the barrel to monitor variations in pore water pressure under negative pressure conditions. It was connected to multi-channel static resistance strain data acquisition instrument using the data transmission line. Finally, the barrel was sealed with a vacuum membrane and sealant. The vacuum pump was connected to the vacuum filtration bottle, and the dewatering process was carried out at a vacuum pressure of −80 kPa. An electronic balance was placed underneath the filter bottle to record the water drainage. The test data were recorded accordingly.

#### Mechanical strength test

**Fig. 5:**
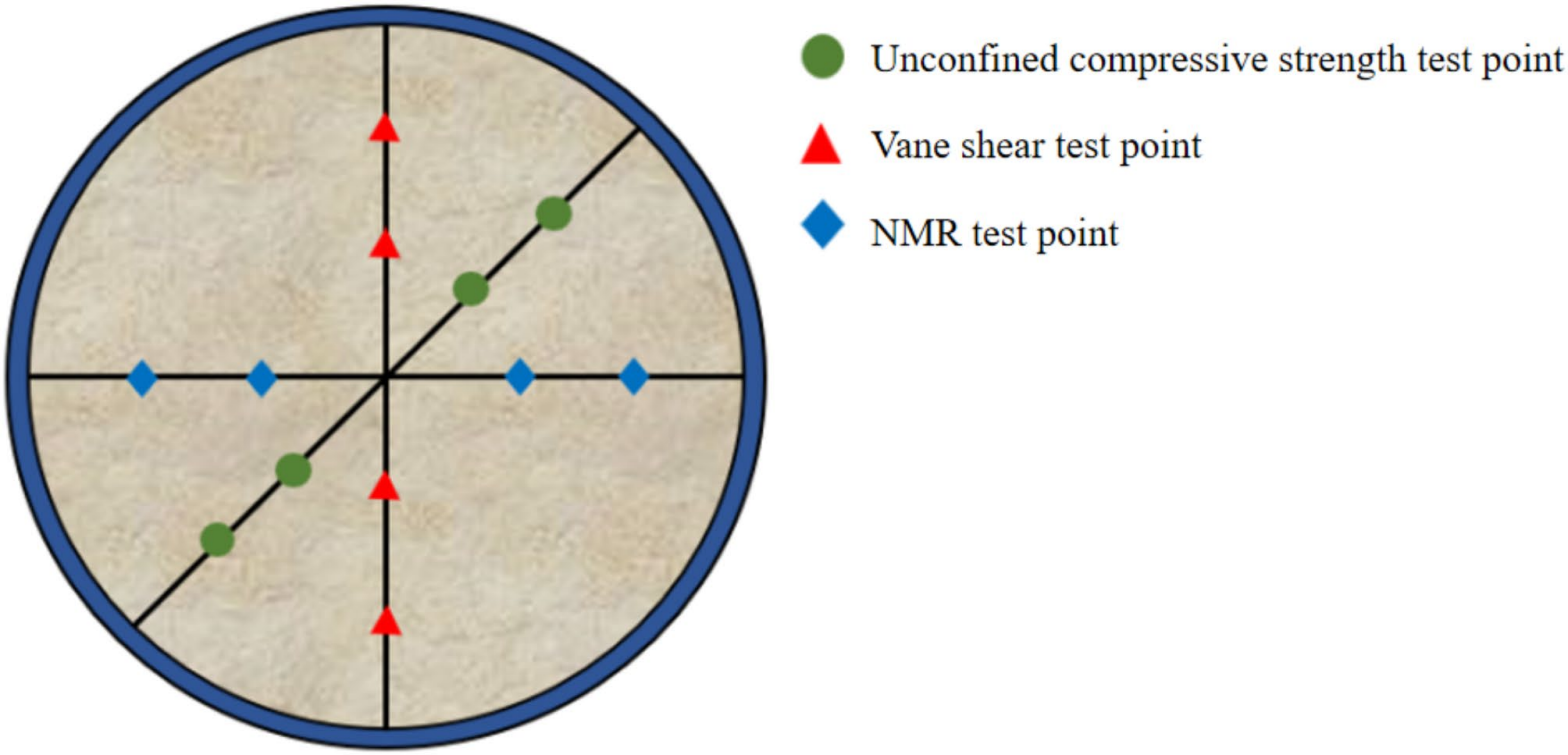
Top view of test points.

After the model test is completed, vane shear strength and unconfined compressive strength of M1-M7 test groups were measured according to the measurement marked in Fig. 5, following *GB/T 50,123-2019 ‘Standard for Geotechnical Testing Methods’*. The loading rate of unconfined compressive strength was set as 1 mm/min.

#### Nuclear magnetic resonance (NMR) test

The pore distribution of samples at 7 days was analyzed using the low-field nuclear magnetic resonance (NMR) MesoMR23-060 H-I, provided by Suzhou Niumag Analytical Instruments Co., Ltd. When measurements are taken with a short echo interval and only water is present in the pores, the surface relaxation effect is typically the main influencing factor, while the impact of other factors is minimal and can usually be neglected. The calculation process can be simplified to Eq. (2)

2 $$\frac{1}{{{T_2}}} \approx {\rho _2}{\left( {\frac{S}{V}} \right)_\rho }={\rho _2}{\left( {\frac{\alpha }{\gamma }} \right)_\rho }$$

where *ρ*_*2*_ represents the transverse relaxation rate, which is related to the physicochemical properties of the soil. S and V refer to the surface area and volume of the pores occupied by water, respectively. *α* is constant at 3, and *γ* represents the pore diameter.

## Determination of formulation of curing agents

### Model adequacy checking

The samples were cured and unconfined compressive tests were subsequently conducted at 7, 14, and 28 days. The test results are shown in Table 3.

**Table 3:** Unconfined compression test results.

No.	7 days (kPa)	14 days (kPa)	28 days (kPa)
1	129	168	228
2	296	336	394
3	268	308	442
4	512	624	1018
5	367	409	465
6	391	472	640
7	233	519	1206
8	452	534	826
9	294	323	1040
10	427	479	762
11	488	594	1022
12	305	382	496
13	262	381	901
14	95	109	115
15	189	306	452
16	227	314	444

The experimental data were analyzed using DesignExpert 10.0.3 to develop regression models and perform analysis of variance (ANOVA). A second-order polynomial model was applied to fit the data and derive the final models. The ANOVA results for the models are presented in Table 4. As shown, the *p*-value of the F-statistic was used to test the hypotheses for both the overall regression model and the individual regression coefficients, with a *p*-value below 0.05 indicating statistical significance.

**Table 4:** Analysis of variance.

Source	*P* value		
	7 days	14 days	28 days
Model	< 0.0001	< 0.0001	< 0.0001
AB	0.0225	0.3193	0.0052
AC	0.0123	0.0033	0.0060
BC	0.3361	0.0113	0.0052
ABC	< 0.0001	0.0025	0.0385
Lack of fit	0.8563	0.9239	0.1160
Regular R^2^	0.9665	0.9621	0.9673
Adjusted R^2^	0.9441	0.9369	0.9455
Predicted R^2^	0.8628	0.8622	0.8519

A, OPC; B, RHA; C. SS.

Non-significant terms were excluded from the model equations for all three curing periods. After removing, the models are shown as Eqs. (3)–(5).

3 $${y_{7d}}=418.81{x_1}+429.34{x_2}+1549.03{x_3} - 1280.66{x_1}{x_2} - 2318.65{x_1}{x_3} - 31252.74{x_2}{x_3}+63341.1{x_1}{x_2}{x_3}$$

4 $${y_{14d}}=490.2{x_1}+51.98{x_2} - 2198.75{x_3} - 646.05{x_1}{x_2}+3858.47{x_1}{x_3} - 12324.19{x_2}{x_3}+32941.1{x_1}{x_2}{x_3}$$

5 $${y_{28d}}=1345.08{x_1}+1332.37{x_2} - 5812.34{x_3} - 4936.03{x_1}{x_2}+766.84{x_1}{x_3} - 11400.11{x_2}{x_3}+42199.6{x_2}{x_2}{x_3}$$

**Fig. 6:**
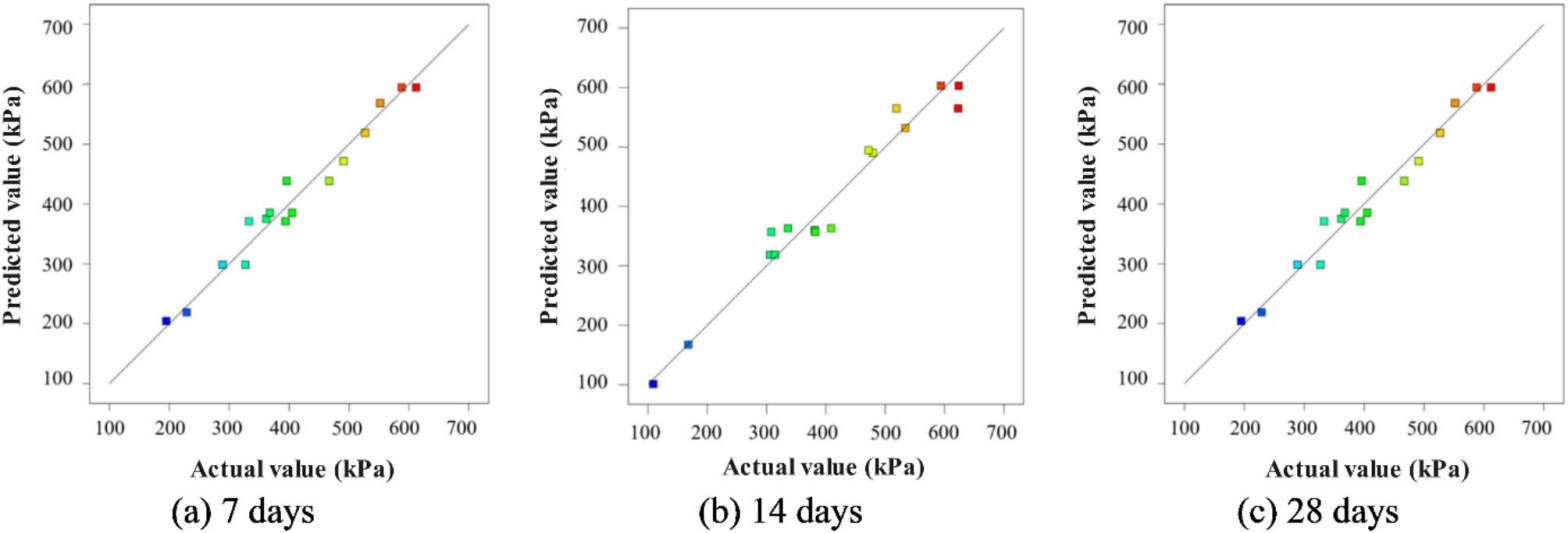
Relationship between predicted and actual unconfined compressive strength.

Figure 6 presents the relationship between the predicted and actual values of unconfined compressive strength. It can be observed that the values are closely aligned, with a slope of 1. The adjusted R^2^ values for the model fits were 0.9665, 0.9621, and 0.9673, respectively, which represents a high level of goodness-of-fit for the models. In conclusion, the three regression models were deemed reasonable approximations of the true functional relationships across the entire range of the investigated variables.

### Interpretation of physical significance

**Fig. 7:**
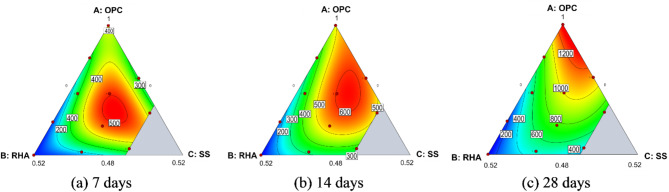
Contour map of unconfined compressive strength.

Response surface contour plots were generated to provide an intuitive analysis of the influence of various factors on compressive strength, as well as the synergistic effects of interactions between factors. The impact of the interactions between the components of the composite curing agent on compressive strength is illustrated in Fig. 7. The shape of the contour plots and the gradient of the axes are directly related to the influence of each factor on the response variable.

Early strength development (7 days) in the sludge is largely attributed to the formation and function of cement hydration products. These products not only interact with soil minerals to form aggregates but also facilitate reactions in which Ca^2+^ ions combine with active silicon and aluminum from the sludge to produce crystalline hydration compounds, thereby establishing the structural framework of the soil^30,31^. The pozzolanic reactivity of RHA is relatively subdued initially, likely due to a low concentration of Ca(OH)_2_. At this phase, a significant portion of Ca^2+^ and OH^−^ ions adsorbs onto the surfaces of RHA particles, thereby weakening the Si−O bonds^32^. Additionally, the high initial water content in the sludge leads to unsatisfactory early performance when using pure cement as the curing agent^12,17^. From a microstructural standpoint, RHA’s porous nature allows it to absorb substantial amounts of water during early hydration^33^. Consequently, the composite curing agent (located near the center of the ternary diagram) exhibits enhanced performance, achieving strengths in excess of 500 kPa.

The highest response values at 28 days (~ 1200) are observed in regions with high OPC content (near the A apex), indicating that OPC, as the primary cementitious material, still plays a crucial role in strength development. As curing progresses, the free water content diminishes, and the water initially adsorbed by the RHA is gradually released^34^. This liberated water further facilitates ongoing cement hydration within the stabilized soil. Additionally, RHA further participates in the pozzolanic reaction upon dissolution. The newly formed C–S–H gel effectively fills the pores within the flocculated soil matrix, significantly reducing porosity and enhancing long-term strength development. However, its optimal dosage must be carefully balanced with OPC and SS to maximize performance.

### Formula optimization and validation

Considering the time constraints of practical construction, a 7-day unconfined compressive strength of the soil exceeding 300 kPa is deemed sufficient to meet the demands of most on-site mechanical construction applications. Hence, the maximum strength of 300 kPa achievable at 7 days was selected with an optimal formula of *x*_*1*_ = 0.53, *x*_*2*_ = 0.32, and *x*_*3*_ = 0.15, representing the proportion of cement, RHA, and sodium silicate, respectively. With this formula, the compressive strengths of the solidified soil at 7, 14, and 28 days are 300 kPa, 394 kPa, and 569 kPa, respectively.

**Fig. 8:**
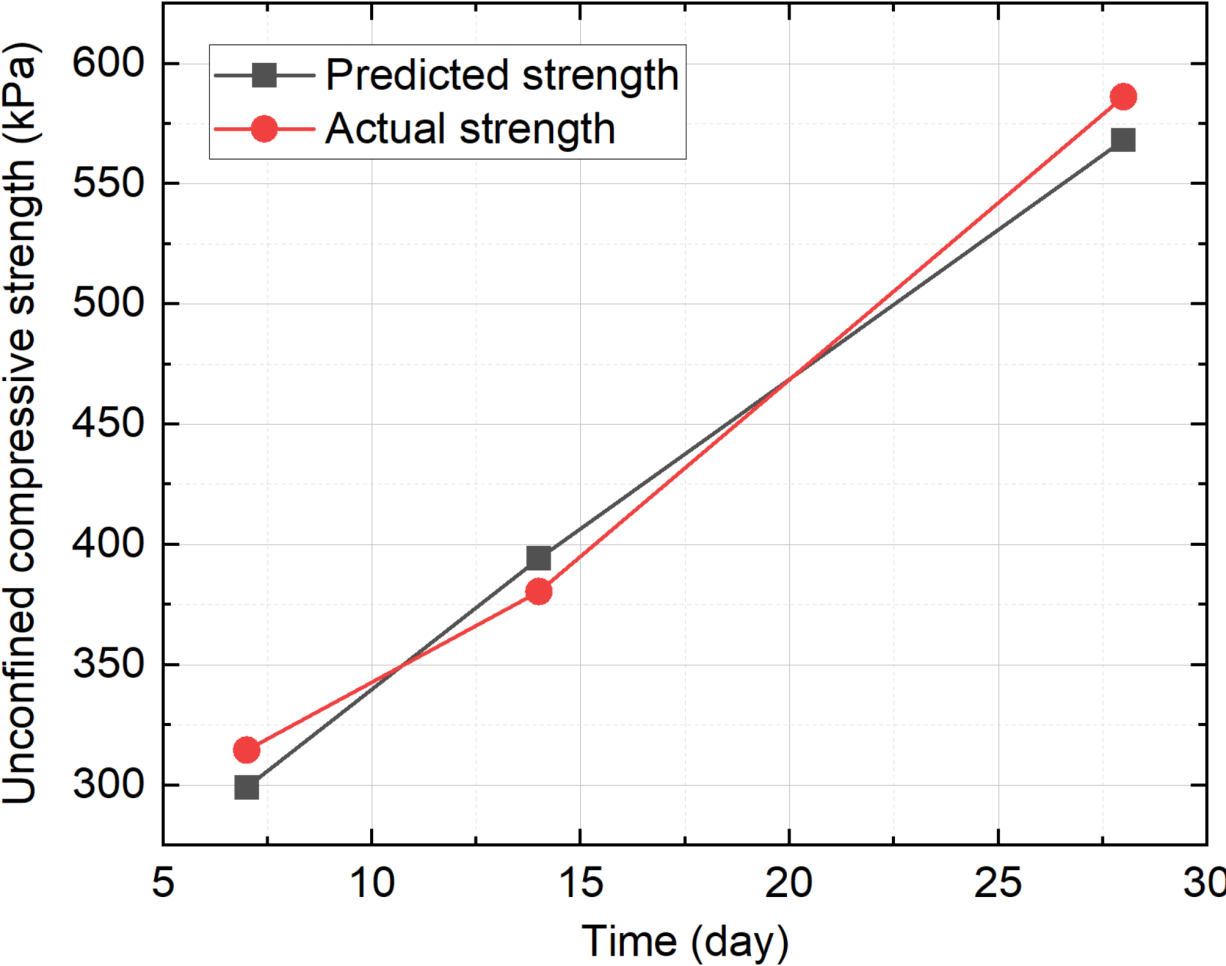
Comparisons between actual and predicted unconfined compressive strength.

Based on the previously calculated mix proportions, new samples were prepared and testes at 7, 14, and 28 days. As presented in Fig. 8, the experimentally measured compressive strengths at 7, 14, and 28 days closely align with the predicted values, demonstrating the accuracy of the model in predicting the strength development over time. Hence, this formular or composite curing agent was selected in subsequent vacuum dewatering experimental studies.

## Vacuum dewatering results and analysis

### Water drainage

**Fig. 9:**
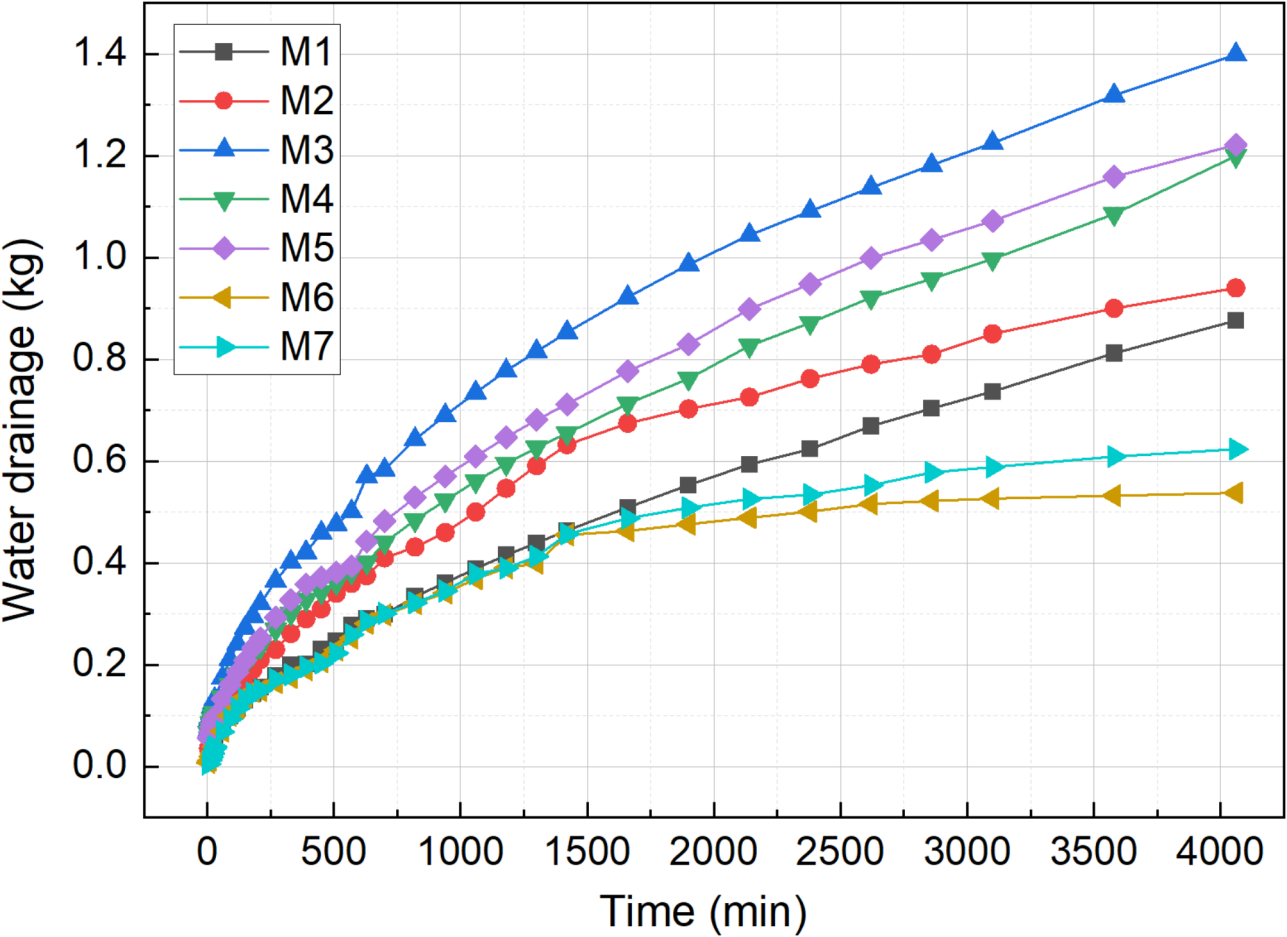
Volume of water drainage with time.

The variation in drainage volume under each treatment scheme is illustrated in Fig. 9. Compared to the M1 group, the drainage volumes of the M2, M3, M4, and M5 groups showed a significant increase by 8.05%, 59.66%, 36.89%, and 39.4%, respectively. Our previous research^35^ shows that as the pH value of the sludge changes, the surface charge of the sludge is neutralized by H^+^, disrupting the stability of the sludge system. Acid treatment of the sludge leads to the dissolution of a large amount of extracellular polymeric substance (EPS), causing microbial cell lysis and the release of bound water, which results in a significant decrease in EPS concentration^36,37^, which has a positive effect on improving the dewatering performance of the sludge^38,39^.

Following pH adjustment, the addition of flocculants (M3, M4 and M5) further promoted the flocculation of the sludge, which increased the available free water for drainage and fully activated the sludge’s drainage potential. In the M6 and M7 groups, 25% cement or 25% composite curing agent were added, respectively. However, the total drainage in these groups was significantly lower. Although the initial setting time of the curing agent was extended by adding Na_4_P_2_O_7_ before testing, analysis showed that sludge dewatering occurred more rapidly within the first 1420 min. Compared with the M1 group, the dewatering volume in the M6 and M7 groups decreased by 38.65% and 28.82%, respectively.

### Surface settlement

**Fig. 10:**
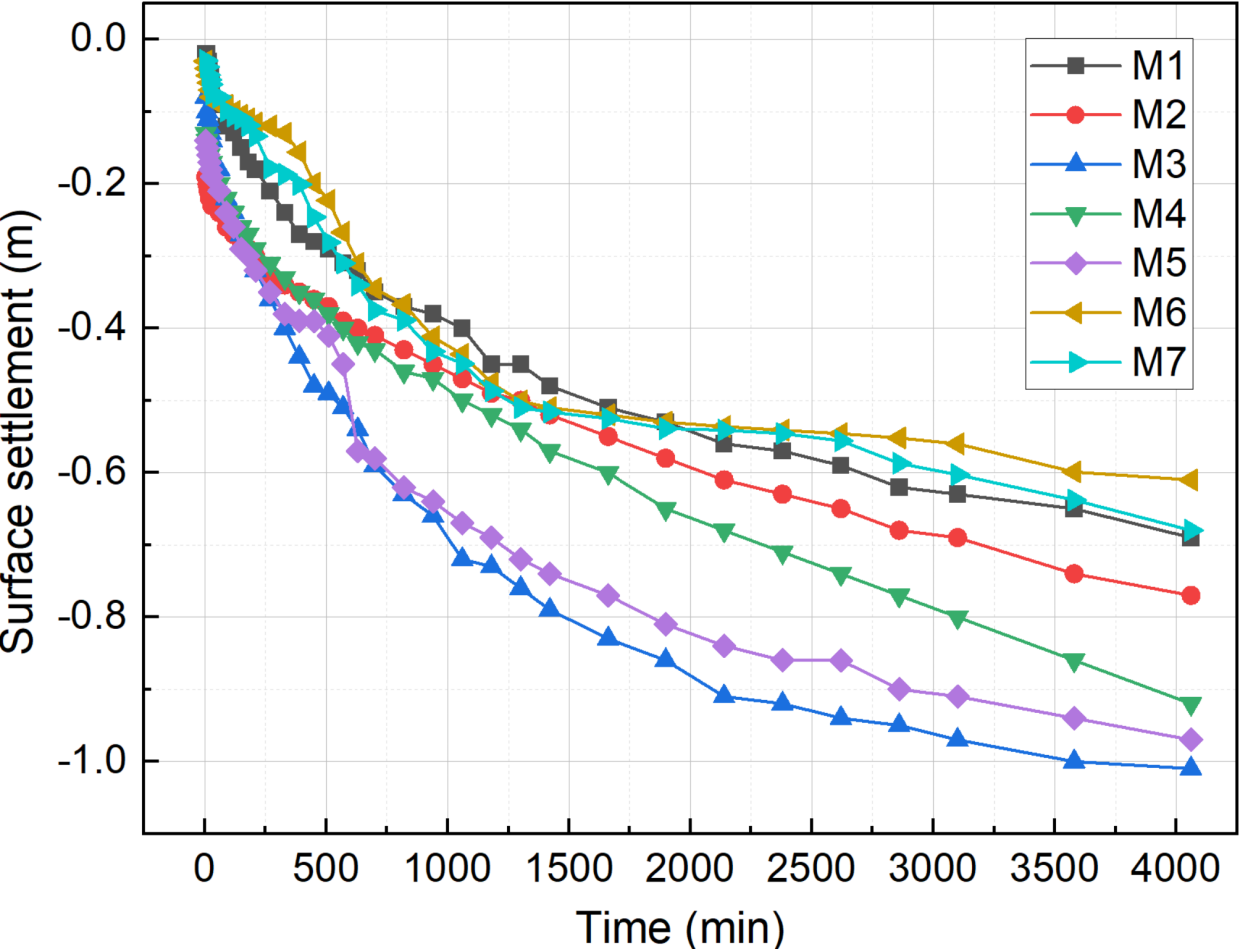
Surface sedimentation with time.

Figure 10 presents the surface settlement of the sludge with time, with the final settlement values following the order: M3 > M5 > M4 > M2 > M1 > M7 > M6, which is consistent with water drainage results. Under the combined influence of pH adjustment, flocculation with vacuum preloading, the M1–M5 groups exhibited rapid soil settlement along with the significant drainage of water from the sludge. The effectiveness of vacuum preloading combined with sole PAC or APAM flocculation was significantly inferior to that of the PAC-APAM composite flocculation system. This superiority can be attributed to the enhanced capacity of composite flocculants to improve soil dewatering compared to individual flocculants, thereby significantly accelerating the sludge drainage rate and leading to a pronounced reduction in surface settlement.

In the M6 and M7 groups, treated with curing agents, settlement rapidly stabilized at approximately 1500 min. The cementitious gel-like compounds produced through chemical reactions enhanced the early compressive strength of the sludge, reinforcing the soil structure and significantly reducing settlement compared to the other test groups.

### Pore water pressure

**Fig. 11:**
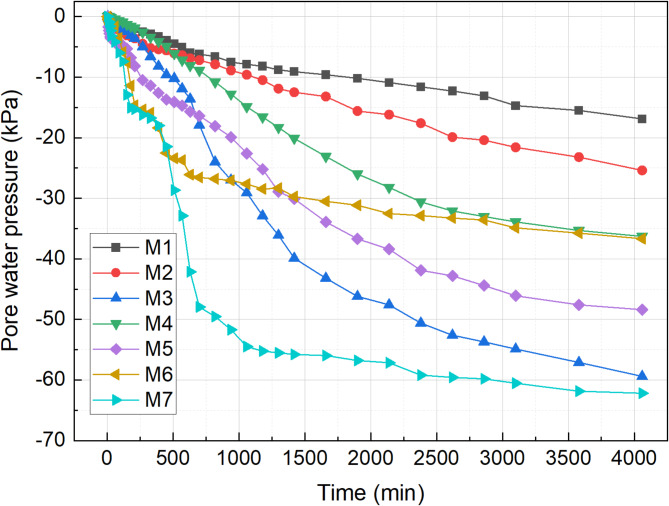
Pore water pressure with time.

The relationship between pore pressure and time for the M1-M7 test groups is illustrated in Fig. 11. Throughout the test, the pore water pressure steadily dissipated due to the application of vacuum preloading. Notably, the M3–M5 groups exhibited a significantly higher dissipation rate compared to the M1 and M2 groups, attributed to the flocculation treatment. Under negative pressure conditions, a pressure differential develops between the soil and its surroundings, causing free water to be expelled through the drainage plate and leading to the dissipation of pore water pressure within the soil. Following pH adjustment combined with flocculation treatment, the sediment experienced more rapid solid-liquid separation. The formation of large, interconnected flocculated aggregates resulted in a more efficient drainage structure, thereby significantly accelerating the dissipation of pore water pressure. Under the combined effect of the flocculant and curing agent, losses associated with the transfer of vacuum pressure within the soil are significantly reduced, thereby enhancing the efficiency of the vacuum preloading treatment. This improvement is also reflected in the notable increases in drainage volume and settlement.

The final pore water pressures of the seven test groups were recorded as − 16.9 kPa, − 25.4 kPa, − 59.4 kPa, − 36.3 kPa, − 48.4 kPa, − 36.68 kPa, and − 62.19 kPa, respectively. Compared to pure cement curing, pore water pressure dissipation in M7, which used a composite curing agent, occurred more rapidly, particularly during the first 1000 min. This could be due to the rapid water absorption capacity of RHA^32^. As the hydration reaction progressed and additional cementitious materials formed, the dissipation rate gradually stabilized, eventually aligning closely with that of the M3 group.

### Vane shear strength

**Fig. 12:**
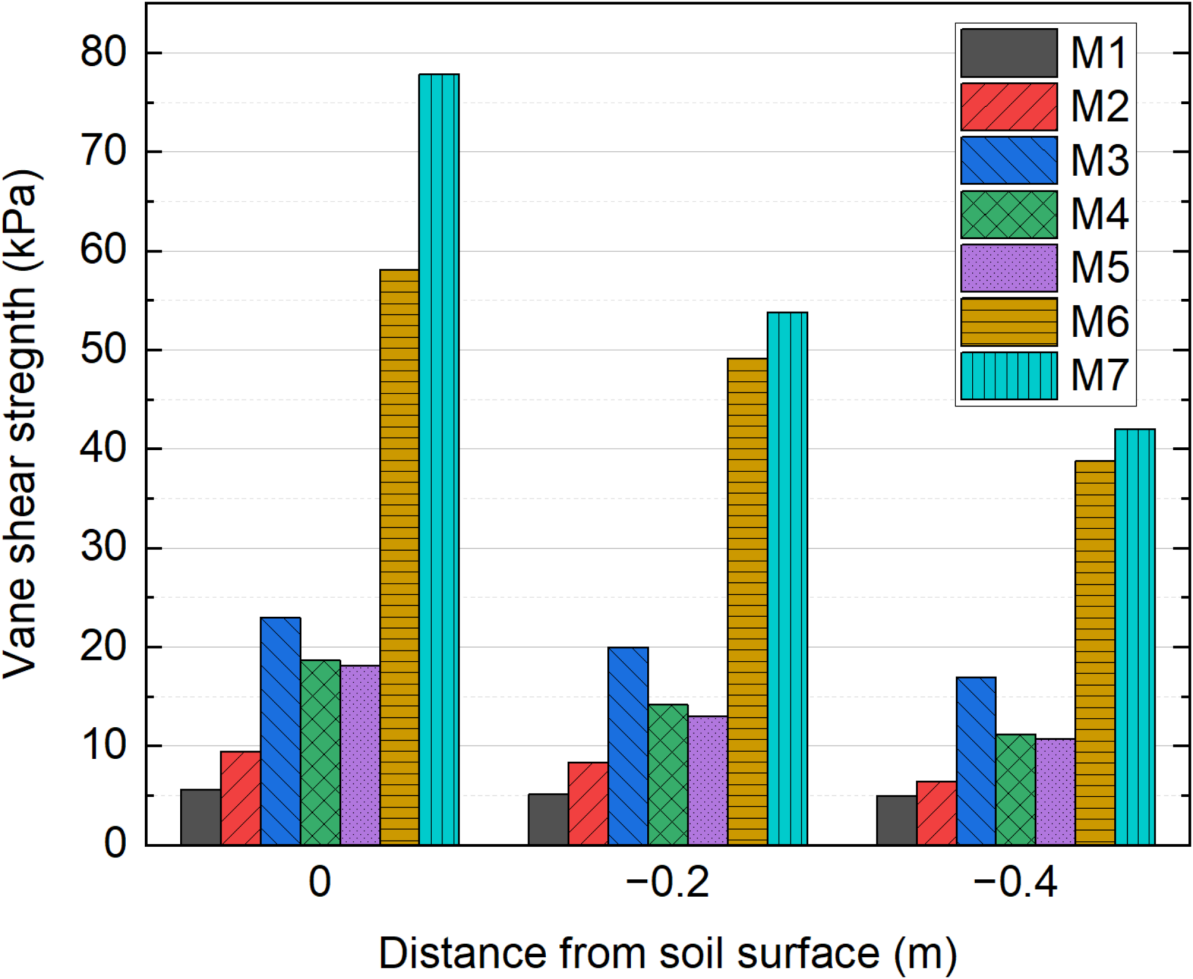
Vane shear strength test results.

Figure 12 illustrates the vane shear strength at varying depths from the soil surface, with mean values compared at 0 m, − 0.2 m, and − 0.4 m. The most significant improvement in vane shear strength was observed at the soil surface following vacuum preloading, with strength gradually decreasing with depth. The average vane shear strength for the M1–M7 groups was 5.4 kPa, 8.33 kPa, 20.03 kPa, 14.2 kPa, 14.57 kPa, 48.67 kPa, and 58 kPa, respectively. Compared to M1, the M2, M3, M4, and M5 groups exhibited shear strength increases of 54.26%, 270.93%, 140.46%, and 170.74%, respectively. Notably, the M6 and M7 groups, treated with curing agents, demonstrated the most substantial improvements, with increases of 801.2% and 974.07%, respectively, highlighting the significant enhancement in soil strength induced by chemical solidification. The enhancement in vane shear strength is primarily driven by the synergistic effect of flocculation and solidification, wherein free water is extracted through drainage plates under negative pressure. Notably, composite flocculant demonstrates a significantly greater efficacy compared to single flocculant, while the incorporation of a curing agent further accelerates the development of shear strength within a short timeframe.

The vane shear strength exhibits a decreasing trend with depth. At greater depths, the sludge experiences reduced efficiency in water drainage or air release due to the resistance from overlying layers, leading to lower vacuum pressure. Additionally, as the flocculated material settles to the bottom, it begins to form the initial structural skeleton. However, as settling continues, the increasing self-weight disrupts the flocculated structure, causing significant variations in the sludge’s composition across different layers. The uneven distribution of sludge, combined with poor air circulation and incomplete water drainage, results in a reduction in strength at these deeper levels.

### Unconfined compressive strength

**Fig. 13:**
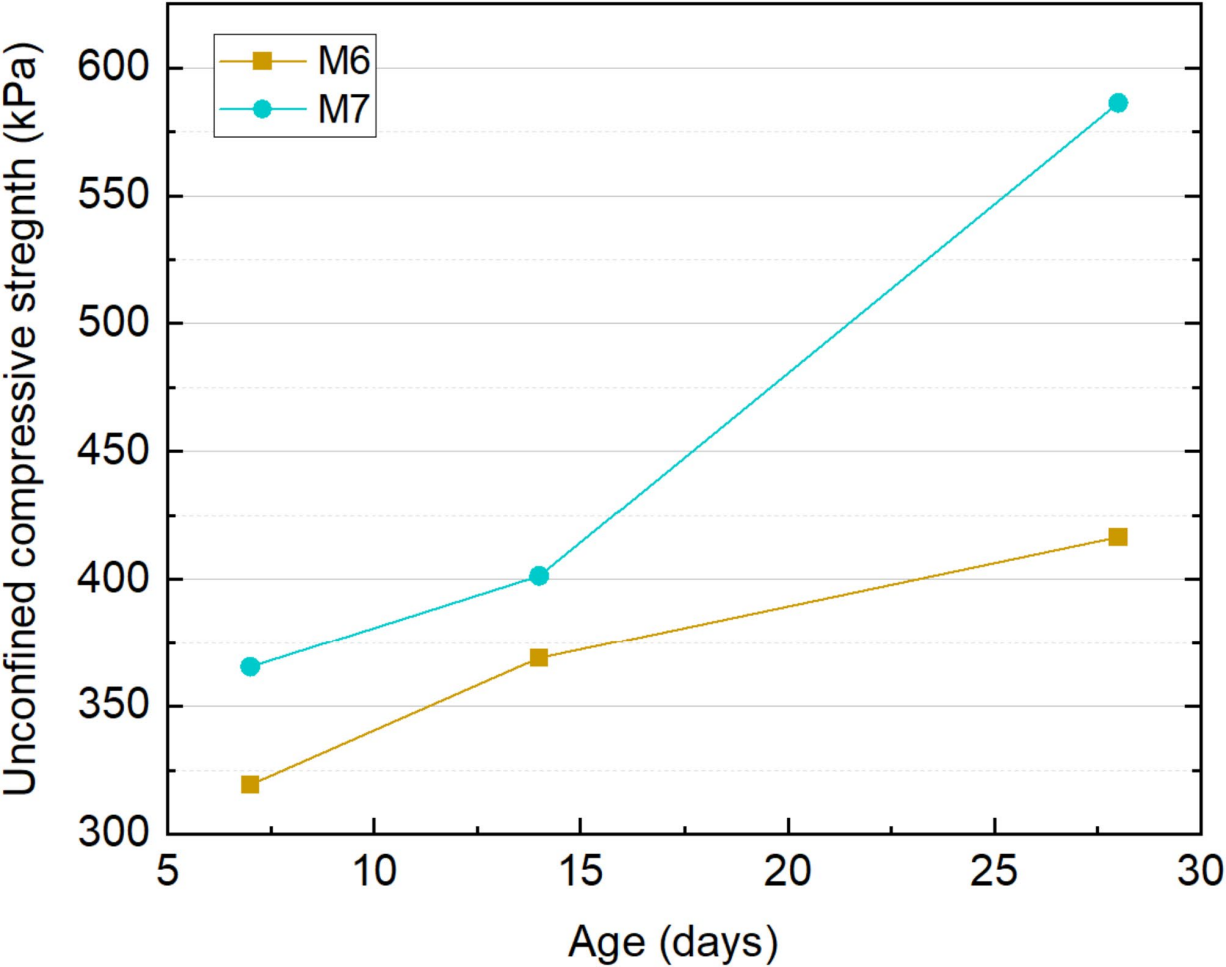
Unconfined compressive strength test results.

Following sampling, the sludge in the M1–M5 test groups exhibited insufficient strength for fabricating unit samples after treatment. Hence, only the M6 and M7 test groups were subjected to unconfined compressive strength testing. Figure 13 illustrates the comparison of unconfined compressive strength between the two groups after a 7-day curing period. Test results show that the compressive strengths of the M6 and M7 groups reached 318 kPa and 365 kPa, respectively. The strength development of M6 was primarily attributed to cement hydration, leading to the formation of a substantial amount of C–S–H gel. In comparison, the composite curing agent in M7 resulted in a significant strength increase at 28 days, indicating a strong latent pozzolanic reaction between the OPC, RHA, and SS at later stages. This late-age strength development demonstrates that FSMV treatment effectively transformed the flowing sludge into solidified soil with substantial strength, thereby creating favorable site conditions for most mechanical construction activities shortly after treatment.

### Nuclear magnetic resonance

**Fig. 14:**
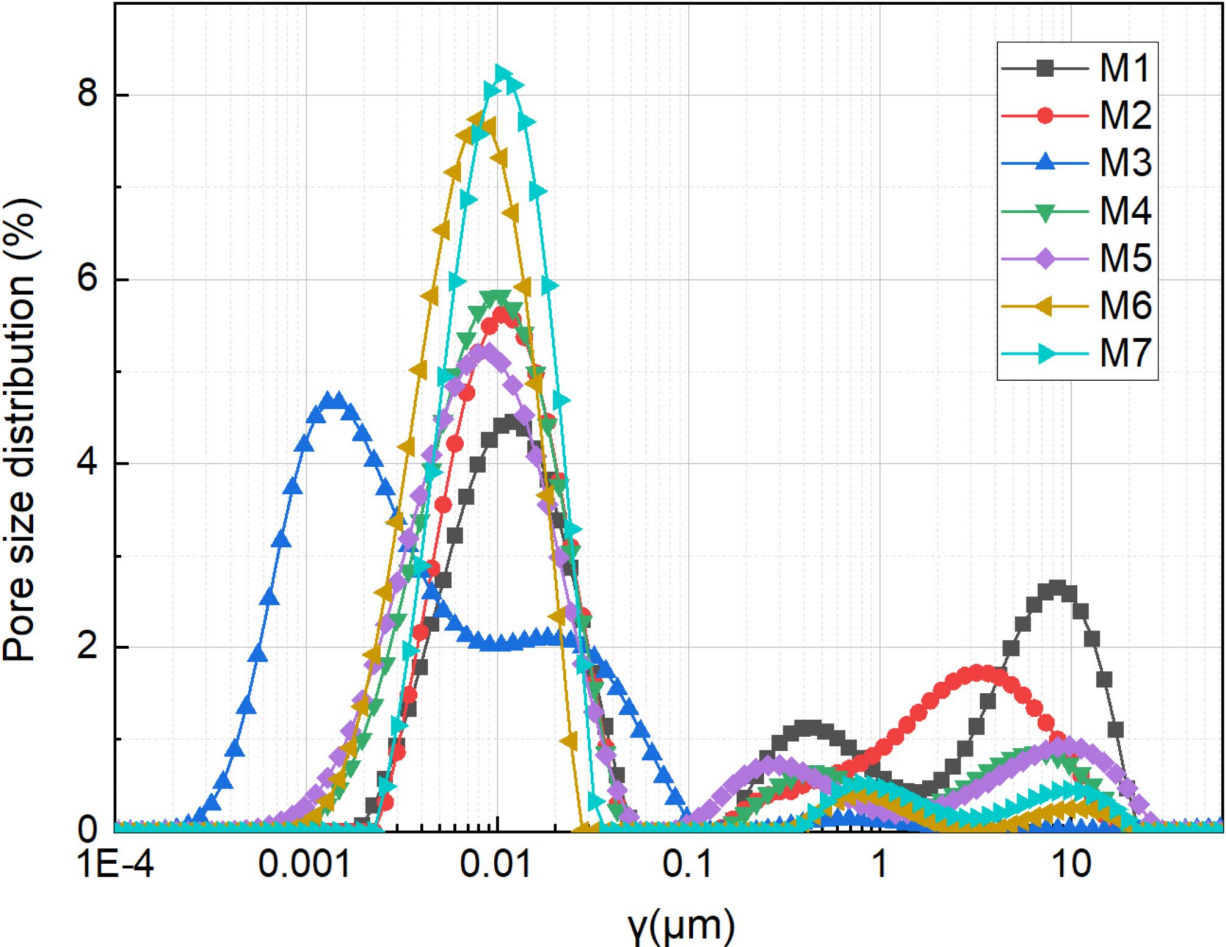
Pore distribution curves.

After the experiment, samples from each group were collected and analyzed using NMR testing. The pore structure of the sludge can be characterized by analyzing the relaxation time of pore water, as presented in Fig. 14. The pore size distribution of the samples spans a wide range, from the nanometre scale (10^−4^ μm) to the micrometre scale (10 μm), effectively characterizing pores across multiple orders of magnitude. This also indicates the presence of a distinct multi-scale pore structure within the samples. M1 and M2 exhibit a broader distribution range, with M1 showing a significant proportion of larger pores in the 1–10 μm range, suggesting higher permeability but a relatively loose structure without pH adjustment. The impact of pH adjustment was relatively modest in changing the pore structure.

Most samples (M3–M7) have pores concentrated around 0.01 μm, predominantly featuring small pores, suggesting sludge dewatering capacity was significantly enhanced through pH adjustment combined with flocculation. The incorporation of flocculant facilitated the formation of efficient drainage channels through electrostatic neutralization and adsorption bridging effects. This minimized vacuum loss during transfer and promoted the effective removal of free water from the sludge. Furthermore, flocculation mitigated clogging during the later stages of vacuum dehydration, further enhancing the efficiency of vacuum preloading. Notably, M3 demonstrates a peak shift towards finer pores (~ 0.001 μm), indicating a higher micropore content and a more compact structure.

The pore size distribution of M7 is similar to that of M6, with a primary peak around 0.01 μm. After the incorporation of a curing agent, hydration reactions generated a substantial amount of C–S–H cementitious material, effectively filling large soil pores, reducing the proportion of macropores, and significantly enhancing soil compactness. Compared to M6, M7 exhibits a higher overall curve amplitude, indicating greater porosity and a more concentrated pore size distribution, suggesting a more uniform internal structure.

### Discussions

This research explores the synergistic effect between flocculation and solidification mechanisms, transforming high-water-content sludge into a cohesive solidified soil structure. The flocculation-solidification process integrates physical sedimentation and consolidation with chemical binding reactions, providing an effective method for stabilizing sludge and enhancing its mechanical properties.

Flocculation plays a crucial role in promoting solid-liquid separation and the subsequent formation of a stable soil skeleton. The polar functional groups in the APAM flocculant enable it to rapidly connect suspended soil particles through adsorption and aggregation, forming larger flocculated structures^40^. Although the clay particles in the sludge carry negative charges, the relatively weak charge neutralization effect does not significantly influence the Zeta potential^41^. However, the polymer chains of APAM can bridge these particles, facilitating the formation of larger flocs and accelerating sedimentation^42^. Similarly, the electro-neutralization effect of PAC accelerates the deposition of sludge, while the adsorption bridging effect of APAM connects fine soil particles in the sludge. The combination of APAM and PAC produces a synergistic effect, improving flocculation efficiency beyond what is achievable with a single flocculant, thereby expediting the sedimentation process and forming a more stable flocculated structure^43,44^.

After pH adjustment, the pore size distribution of samples treated flocculation primarily concentrates around 0.01 μm, indicating that the soil mainly contains fine pores. Notably, samples flocculated with PAC-APAM exhibit a shift in the peak pore size distribution towards approximately 0.001 μm, indicating an increase in micropore content and a more compact structure. This pore refinement contributes to water discharge. Despite the effectiveness of flocculation, it alone cannot provide sufficient compressive strength to the sludge.

With the addition of a curing agent, the sludge can achieve an early unconfined compressive strength over 300 kPa. During the flocculation-solidification-vacuum preloading process, the hydration reaction of cementitious materials consumes a significant amount of free water, leading to the formation of a large quantity of C-S-H gel and other cementitious products. These products fill soil pores, establishing a spatial network that enhances the strength of the solidified soil. Furthermore, the hydrolysis of PAC releases Al^3+^ ions, which react with Ca(OH)_2_ to form ettringite^10^. This hydration product plays a crucial role in improving the structural integrity and mechanical strength of the solidified soil by reinforcing the network structure among soil particles. In the composite curing agent, not only does the hydration reaction of OPC occur, but the additional pozzolanic reaction of RHA further enhances the formation of cementitious compounds by reacting with Ca(OH)_2_ to produce extra C-S-H gel and other binding phases. This dual mechanism results in a more uniform and densely compacted soil structure, directly contributing to the observed improvement in compressive strength.

Overall, the FSVP treatment enables the rapid stabilization of high-moisture-content sludge. The integration of chemical binding reactions and physical sedimentation offers an effective mechanism for transforming sludge into a durable and stable material. This approach holds significant promise for sludge treatment applications in geotechnical and environmental engineering.

## Conclusions

This study investigates the formulation of curing agents using the response surface methodology. After determining the optimal formulation, the synergistic effects of flocculation, solidification, and vacuum preloading were further explored. Based on the analysis of the test results, the following conclusions were drawn:

- Considering the strength development of flocculated and stabilized soil at different curing ages, the optimal mix ratio of the composite stabilizer was determined to be 53% cement, 32% rice husk ash, and 15% sodium silicate. This mix ratio balances both the early and later strength of the flocculated and stabilized soil, meeting the objectives of cement usage reduction and practical engineering requirements.
- After pH adjustment, the flocculation under vacuum preloading performs excellently in the rapid dewatering treatment of sludge. In particular, the composite application of APAM and PAC as flocculants results in a peak shift toward finer pores (~ 0.001 μm), indicating an increased micropore content and a more compact structure.
- The addition of a curing agent influences the duration of the vacuum preloading process by refining the pore sizes, initially leading to poor water drainage and settlement. A higher overall curve amplitude can be seen on the pore distribution curve of samples using composite curing agent (M7), suggesting more concentrated pore size distribution and a more uniform internal structure.
- The application of a composite curing agent promotes the hydration and pozzolanic reaction, particularly benefiting strength development at later ages. The formation of cementitious product and consumption of water result in an increase in both vane shear strength and unconfined compressive strength.

## Data Availability

The original contributions presented in the study are included in the article, further inquiries can be directed to the corresponding author.
